# A forensic aspect of age characteristics of dentine using transversal microradiography: a case report

**DOI:** 10.1186/1757-1626-2-4

**Published:** 2009-01-02

**Authors:** Leonidas Vasiliadis, Christos Stavrianos, Panagiotis Kafas

**Affiliations:** 1 Department of Endodontics (Forensic Dentistry), School of Dentistry, Aristotle University, Thessalonica, Greece; 2 Department of Oral Surgery and Radiology, School of Dentistry, Aristotle University, Thessalonica, Greece

## Abstract

**Background:**

Translucency of dentine is the result of occlusion of the corresponding dentinal tubules by a mineral substance which has a refractive index similar to that of the rest of the dentine.

**Case presentation:**

This case report describes the microradiographic features of an upper cadaveric canine. Transverse microradiograph is one of the methods assessing apical dentine translucency for various dental and medical reasons.

**Conclusion:**

Estimation of age using teeth structures may be of primary value in forensic dentistry, especially when soft tissues are severely destructed.

## Background

Microradiography has been employed in dental research to measure mineral distribution in hard tissues such as bone, enamel and dentine [1].

It is shown that for wavelengths between 0.5 A° and 3 A° that is for the soft x-rays, the mass-absorption coefficient for the organic fraction of the mineralized tissues is only about 1/10 of that of the apatite. Applying these findings to the dentine of human teeth and taking into consideration that the organic content of dentine is less than 1/4 of the total by weight, it is stated that the absorption of the organic matrix of the dentine is less than 2.5% of the total absorption of the dentine [2]. Thus, practically, microradiography of the dentine is the study of the mineral, inorganic, phase of the dentine.

This case report emphasizes the possible use of microradiography in forensics. It was our interest to evaluate the dentine characteristics of age using microradiography of a cadaveric canine tooth.

## Case report

A cadaveric upper right canine was selected for estimation of age in forensics. The individual died at 59 years of age. The investigators used an x-ray generator supplied with a fine tube. The radiation emission performed at 20 kv and 30 mA. A nickel filtered copper K alpha monochromatic radiation through a beryllium window used. The target-film distance was measured at 26 cm. The specimen was mounted on spectroscopic plate type 649-0, in the cassettes and held in position with a stretched aluminium foil 25 μm thick. The specimen section (< 55 μm) of the apical third was then subjected to x-ray for 45 min. The microradiographic plate was developed in HRP developer for 5 min, washed in distilled water, rinsed in stop bath (5% solution, per volume, glacial acetic acid) for 30 sec, washed again in distilled water and fixed in two baths for 5 min in each bath. After fixation the plates were washed for 30 min in cold water, rinsed in wetting agent and hung to dry. The microradiographic plate was mounted on microscope slide with D.P.X. mountant and a cover slip was applied using the same mountant.

**Figure 1:**
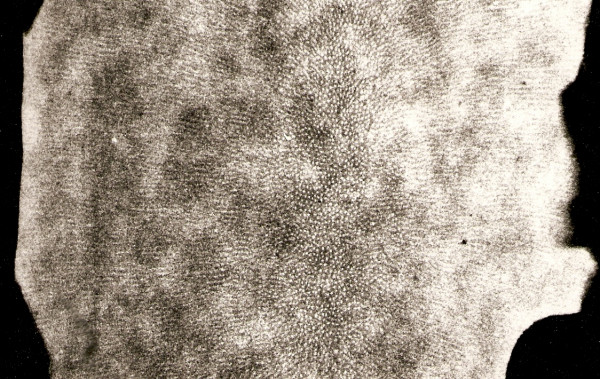
**Microradiograph of the apical third of root dentine assessed by transmitted light microscopy (mag. × 125)**.

**Figure 2:**
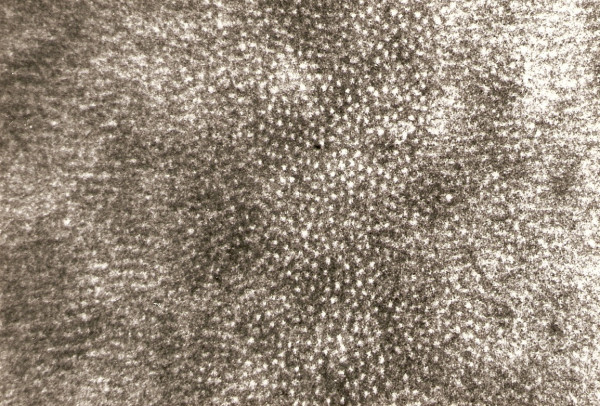
**Figure-1 in higher magnification showing the advanced stage of tubular occlusion due to the superimposition of the x-ray image (mag. × 315)**.

The advanced stage of tubular occlusion was seen clearly (Figure. 1). Therefore microradiography indicated the deposition of the minerals and based on the variations in the x-ray absorption this material. This may happen due to superimposition of x-ray (Figure. 2). The possible influence of age on apical dentine structure using microradiography, were highly occluded tubules and superimposition of the peritubular dentine.

## Discussion

Basically, the study of the microstructure of translucent dentine may be performed using light microscopy, microradiography and SEM examination [3]. The microradiographic technique may be divided into two assessment types. Firstly the ground, longitudinal sections of about 250 μm thick, in order to establish the relationship between the translucent zones and their x-ray absorption [4]. The microradiographs and the original sections may be compared using the enlarger technique. Secondly, the polished transverse sections, 35–55 μm thick, in order to locate the deposition of the extra minerals, if any, and to compare their degree of x-ray absorption with that of the rest of the dentine and to establish how closely packed are the crystals of the deposited minerals [5]. In our case the latter technique was used showing the advanced tubular occlusion with the associated deposition of minerals.

The zones which appeared translucent by optical examination, showed an increased x-ray absorption in the microradiographs. Occasionally islands of completely occluded tubules were seen surrounded by patent tubules. Furthermore, tubule-free (that is neither closed nor patent tubules were detectable) areas were seen near the root canal in sections in which tubules were present anywhere else. The original diameters of the lumens of the completely or partially occluded tubules (the diameter of the circle formed by the line of junction between the intertubular dentine and the smooth material in the tubule) was not noticeable different from that of adjacent patent tubules. As already mentioned, no predentine layer was seen by microradiography at the pulpal ends of the translucent zones of root dentine. This might have been the result of sectioning, grinding, and polishing of the specimen.

The age changes of the root are associated with the reduction of the number of the sub-odontoblastic pulpal blood vessels with increase age. Another sound explanation is that the odontoblasts corresponding to the translucent areas of dentine may be less active than the rest of the odontoblasts [3]. Despite the reduction of the number of the odontoblasts and their blood supply with age, the corresponding part of the dentine does become translucent with age and furthermore at an increasing rate.

Concluding the prementioned features may be used additionally to the well known measures of forensic dentistry. The discussed features of canine microradiography may be used in forensics as a guide of 59 years-old tooth, for estimation of victim's age. Further studies are required for validation of these findings. Furthermore analyses of teeth microradiography seem to be essential for other age groups.

## Consent

Written informed consent was obtained from the patient for publication of this case report and accompanying images. A copy of the written consent is available for review by the Editor-in-Chief of this journal.

## Competing interests

The authors declare that they have no competing interests.

## Authors' contributions

CS, LV and PK were major contributors in assessing the case and writing the manuscript. All authors read and approved the final manuscript.
